# Visual Deprivation’s Impact on Dynamic Posture Control of Trunk: A Comprehensive Sensing Information Analysis of Neurophysiological Mechanisms

**DOI:** 10.3390/s24175849

**Published:** 2024-09-09

**Authors:** Anna Sasaki, Honoka Nagae, Yukio Furusaka, Kei Yasukawa, Hayato Shigetoh, Takayuki Kodama, Junya Miyazaki

**Affiliations:** Department of Physical Therapy, Faculty of Health Science, Kyoto Tachibana University, 34 Yamada-cho, Oyake, Yamashina-ku, Kyoto 607-8175, Japan; a903021088@st.tachibana-u.ac.jp (A.S.); a903021121@st.tachibana-u.ac.jp (H.N.); a903021154@st.tachibana-u.ac.jp (Y.F.); a903021184@st.tachibana-u.ac.jp (K.Y.); kodama-t@tachibana-u.ac.jp (T.K.); j-miyazaki@tachibana-u.ac.jp (J.M.)

**Keywords:** visual information, posture control, random forest

## Abstract

Visual information affects static postural control, but how it affects dynamic postural control still needs to be fully understood. This study investigated the effect of proprioception weighting, influenced by the presence or absence of visual information, on dynamic posture control during voluntary trunk movements. We recorded trunk movement angle and angular velocity, center of pressure (COP), electromyographic, and electroencephalography signals from 35 healthy young adults performing a standing trunk flexion–extension task under two conditions (Vision and No-Vision). A random forest analysis identified the 10 most important variables for classifying the conditions, followed by a Wilcoxon signed-rank test. The results showed lower maximum forward COP displacement and trunk flexion angle, and faster maximum flexion angular velocity in the No-Vision condition. Additionally, the alpha/beta ratio of the POz during the switch phase was higher in the No-Vision condition. These findings suggest that visual deprivation affects cognitive- and sensory-integration-related brain regions during movement phases, indicating that sensory re-weighting due to visual deprivation impacts motor control. The effects of visual deprivation on motor control may be used for evaluation and therapeutic interventions in the future.

## 1. Introduction

Posture control is classified into two types: static posture control, which involves maintaining posture without voluntary displacement of the center of mass (COM), and dynamic posture control, which involves maintaining posture during movements that involve voluntary displacement of the COM. Posture control factors are usually classified into biomechanical constraints, spatial orientation, movement strategy, dynamic control, sensory strategy, and cognitive processing [1]. Posture control has been quantified using various objective indicators, such as movement angle, angular velocity, muscle activity, movement time, and center of pressure (COP) [2,3,4].

Human posture control utilizes two movement strategies: anticipatory postural adjustments (APA) and compensatory postural adjustments (CPA) [5]. The APA is a posture control strategy that minimizes the displacement of the COM in anticipation of disturbances or intentional movement activation. It is also called feedforward control. Conversely, CPA is a posture control strategy that compensates for disturbances and is referred to as feedback control. One example of APA is that when raising one upper limb from a standing position to a horizontal position as quickly as possible, muscle activity appears in the ipsilateral biceps femoris and contralateral erector spinae muscle before the deltoid muscle, which is the primary muscle responsible for the movement [6]. Additionally, psychological factors have been reported to affect movement strategy. Anticipation and fear of pain associated with movement have been found to prolong the switch phase duration of trunk flexion–extension movement [2] and the APA phase duration [4].

Humans predict movement results before performing movements, and the comparator model has been proposed as a mechanism for obtaining a sense of agency when actual results align with predictions [7]. This model makes inferences using information about movement and sensory input observed during movement planning and execution. Sensory information is a critical factor in human posture control, and stable posture is achieved through the integration and processing of sensory information, including vision, vestibular sensory information, and somatosensory perception, by the central nervous system. Sensorimotor cues are utilized in movement control, and cognitive cues such as attention and decision-making also affect movement control [8,9]. This integration strategy can flexibly change according to the individual situation. Sensory weighting is also utilized in posture control assessment, with differences in visual information reflecting the influence of proprioception [10,11]. Previous research has suggested that, in posture control with the eyes closed, there are individuals with a higher weighting of somatosensory input from the lower limbs and those with a relatively high weighting of vestibular sensory input for gravity reference [12]. In addition, when focusing on posture control in closed-eye conditions, COP displacement was reported to decrease in the APA phase, and muscle activity increased compared to open-eye conditions [11]. Therefore, in environments where visual information cannot be accurately used as feedback for body position, the weighting of vision decreases and the weighting of other senses increases, which, it is suggested, influences objective posture control indicators.

Many studies have examined static posture control in standing posture maintenance with and without visual information. However, the influence of visual information on dynamic posture control during voluntary trunk movements is yet to be clarified. It is suggested that sensory control strategies rely more on somatosensory information than on vestibular sensory information, depending on the presence or absence of visual information [13]. By clarifying the influence of sensory re-weighting, including proprioception, due to visual information on dynamic posture control, it becomes possible to evaluate the effect of proprioception on dynamic posture control in individuals with proprioceptive disorders. Additionally, investigating neurophysiological mechanisms, including brain activity, enables the elucidation of pathophysiological mechanisms of dynamic posture control in proprioceptive disorders. Therefore, this study aimed to investigate the effect of sensory re-weighting due to the presence or absence of visual information on dynamic posture control in trunk movements and to examine neurophysiological mechanisms through brain activity analysis.

The hypotheses of this study were as follows: (1) In conditions without visual information, changes in proprioception weighting increase somatosensory input weighting, leading to muscle stiffness, reduced movement maximum angle, decreased angular velocity, and reduced COP–anterior–posterior (COP-AP) displacement. Additionally, considering that in brain regions related to sensorimotor activity, it is suggested that alpha-wave activity reflects visual processing and visuospatial attention and contributes to inhibition mechanisms in the ipsilateral sensorimotor cortex [14]. In contrast, beta-wave activity regulates the disinhibition of regions involved in movement [14]. Therefore, (2) in conditions without visual information, the lack of attention resources through vision increases cortical activity due to cognitive and sensory integration, causing the modulation of alpha- and beta-wave activity.

## 2. Materials and Methods

### 2.1. Participants

The participants comprised 35 healthy young adults (21.6 ± 1.2 years old, 22 males and 13 females). The exclusion criteria included those with orthopedic conditions or neurological symptoms affecting the lumbar spine, hip joints, or knee joints within the last two weeks. This study was approved by the Ethics Committee of Kyoto Tachibana University (approval number: 2024-09), and written informed consent was obtained from the patients.

### 2.2. Study Procedure

**Figure 1:**
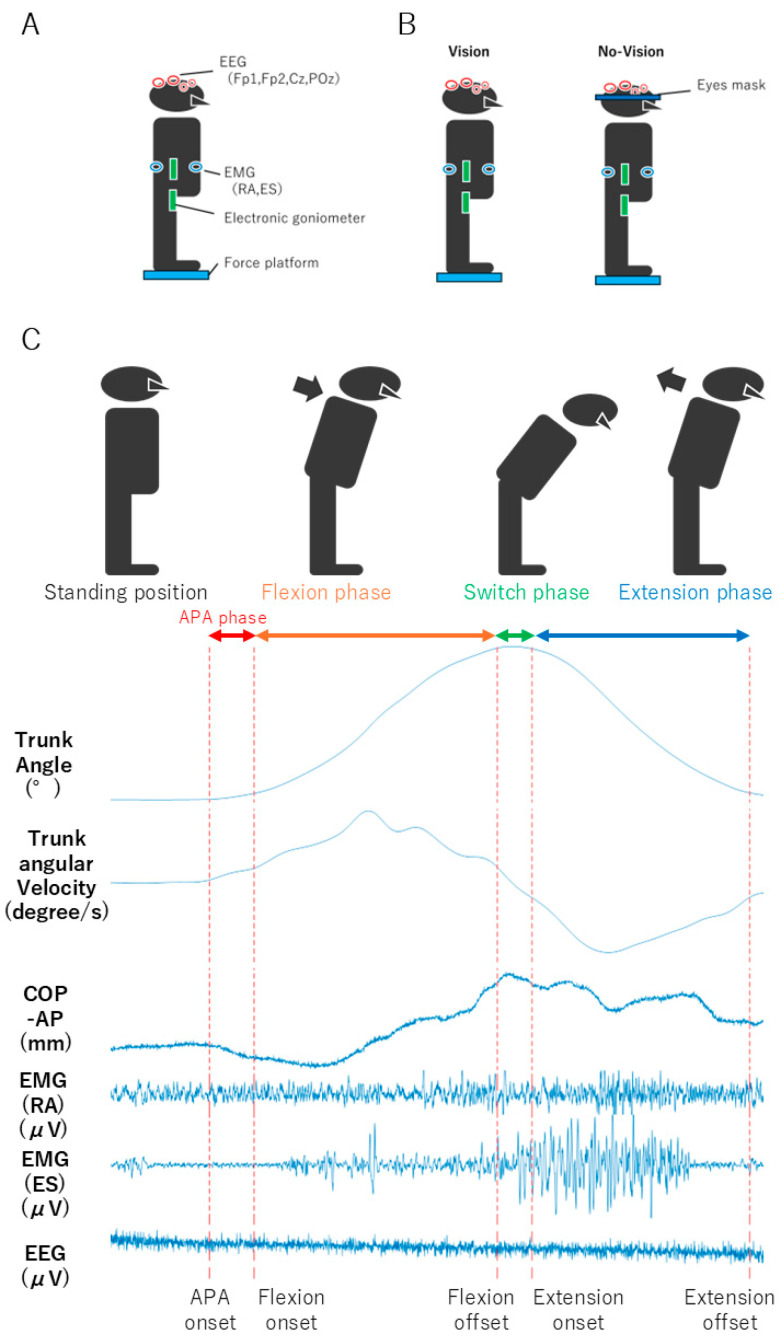
Experimental Environment Setup: (**A**) Research equipment and attachment locations. The two green rectangles represent the two arms of the electronic goniometer. (**B**) Condition setup. In the No-Vision condition, participants wore an eye mask to deprive vision. (**C**) The curves are from one repetition of one subject’s movement tasks and a time series of measurement indicators. The APA, flexion, switch, and extension phases are classified based on angular velocity and COP-AP baseline values to analyze each measurement indicator. EEG: electroencephalography; EMG: electromyography (Fp1: left side prefrontal, Fp2: right side prefrontal, Cz: center of the parietal, POz: back center of the parietal); RA: rectus abdominis; ES: erector spinae; COP: center of pressure; AP: anterior–posterior; APA: anticipatory postural adjustments.

Participants performed the experiments in a fixed order: condition with visual information (Vision condition) first, followed by condition without visual information (No-Vision condition). The Vision condition involved open eyes. In the No-Vision condition, participants wore an eye mask but kept their eyes open to avoid the impact of closed eyes on brain waves. Standing trunk flexion–extension tasks, movement analysis, COP-AP, electroencephalography (EEG), and electromyography (EMG) were measured simultaneously (Figure 1). The measurement times of the devices were synchronized.

### 2.3. Movement Tasks

The standing trunk flexion–extension movement was performed to the maximum extent of lumbar flexion (Figure 1). Then, the lumbar spine was extended backward to return to an upright posture [4]. According to previous research, participants followed the movement procedure and were instructed to move as large and fast as possible. The standing trunk flexion–extension movement included five practice repetitions and three main trials [4].

### 2.4. Movement and COP Analysis

We measured movement angle/angular velocity and COP to identify the impact of visual sensory information on trunk dynamic posture control. In addition, to further clarify each movement phase’s characteristics, we analyzed each phase separately. COP was measured using a force platform (Biosignalplux, Plux, Inc., Lisbon, Portugal). The movement angle was measured using an electronic goniometer (Biosignalplux, Plux, Inc., Lisbon, Portugal). The COP and angle signals were acquired at a sampling frequency of 1000 Hz and filtered using a 4th-order Butterworth filter with a cutoff frequency of 6 Hz. The electronic goniometer was attached to the side of the trunk and measured trunk motion angles based on the height of the 12th spinous process (T12) to the second lumbar spinous process (L2) for the first arm placement, and on the first sacral spinous process (S1) to the third sacral spinous process (S3) for the second arm placement. Based on the measured movement angles, angular velocity was calculated.

In this study, the movement phases were classified based on angular velocity. The flexion phase was defined as the period from when the flexion angular velocity exceeded 15°/s until it peaked and fell below 15°/s. The switch phase was defined as the period from when the flexion direction fell below 15°/s until the extension movement began (15°/s or more in the extension direction). The extension phase was defined as the period from when the angular velocity toward extension exceeded 15°/s until it peaked and fell below 15°/s [4]. Additionally, using previous research as a reference, the start of the COP-AP displacement was defined as 5% of the peak speed of the anterior–posterior COP-AP displacement, and the APA phase was defined as the period from the start of the COP-AP displacement to the start of flexion movement [15]. The maximum COP-AP displacement during the APA phase and the maximum displacement during flexion movement were calculated as COP indicators.

### 2.5. Electromyography

We measured muscle activity to identify the impact of visual sensory information on muscle stiffness. Muscle activity was measured using an EMG sensor (Biosignalplux, Plux, Inc., Lisbon, Portugal). The sampling frequency was set to 1000 Hz, and the Butterworth filter was set to 10–400 Hz to remove noise and ensure that the EMG signal represented true muscle activity. Following filtering, the EMG signal was rectified. Electrodes were placed on the left side of the agonist and antagonist muscles, the erector spinae (ES), and the rectus abdominis (RA) muscle, to measure muscle activity during standing trunk flexion–extension movements. The RA electrode was located 3 cm to the side of the navel, and the ES electrode was placed 3 cm to the side of the third lumbar spinous process following the level of the Jacoby line [3,16]. The reference electrode was attached to the radial styloid process. In this study’s analysis, muscle activity normalization was performed by measuring and using the average muscle activity during 10 s of standing still before the start of the movement task [17]. Additionally, we performed signal rectification to further process the EMG data, which helps in emphasizing true muscle activations. We also conducted a visual inspection of the EMG signals to ensure that the identified peaks corresponded to true muscle activity and not artefacts.

This study calculated the mean muscle activity of the RA and ES in each motion phase to characterize the overall motion phase. The co-contraction index (CCI) of the RA and ES was calculated using the following formula, based on previous research [18]:

CCI=2∗EMGlow/(EMGlow+EMGhigh)

In the CCI formula, EMGlow represents the muscle with lower muscle activity, and EMGhigh represents the muscle with higher muscle activity. In this study, the CCI was calculated for each data sample, and the mean CCI was calculated for each motion phase. A high CCI value indicates strong co-contraction between the RA and ES muscles, suggesting enhanced stability and control during the movement. Conversely, a low CCI value indicates weak co-contraction.

### 2.6. Electroencephalography

We measured EEG to identify the impact of visual sensory information on brain activity in brain regions related to sensorimotor activity. EEG was measured using an EEG sensor (biosignalplux, Creact). Based on the international 10–20 system, the cognition and sensory integration regions, including Fp1, Fp2, Cz, and POz, were measured. The frontal regions, Fp1 and Fp2, which correspond to the prefrontal cortex, were selected because they reflect brain functions related to cognition and emotion [19]. Cz and POz were selected for their involvement in spatial information processing and somatosensory integration in the parietal lobe [20]. The POz region corresponds to the superior parietal lobule. The sampling frequency was set to 1000 Hz. The reference electrode was attached to the right earlobe. The electrode attachment site was cleaned, and the sensors were attached following the biosignalplux measurement manual. Additionally, to minimize arousal’s impact on EEG, participants were instructed to obtain enough sleep the night before the measurement and avoid consuming alcohol and caffeine. The measurement was conducted in a quiet environment to minimize the impact on EEG.

In EEG data analysis, frequency filtering with a Butterworth filter of 1–30 Hz and noise processing by independent component analysis were applied to the measurement data. A wavelet analysis (Morlet wavelet) was performed on the APA, flexion, switch, and extension phases. For each phase, the power spectral density was computed within the theta (4–7 Hz), alpha (8–12 Hz), and beta (13–30 Hz) frequency bands. The power values were then naturally log-transformed using the natural logarithm to normalize their distribution. The alpha/beta (α/β) ratio was calculated by dividing the log-transformed alpha power by the log-transformed beta power for each phase. Theta waves were analyzed to verify whether fatigue and concentration were affected due to conditions and the number of measurements, and no impact was observed. For Fp1 and Fp2, the average was calculated for use as an indicator of EEG activity in the prefrontal cortex region (Fp). The average of Fp1 and Fp2 were determined after all other computations.

### 2.7. Statistical Analysis

This study analyzed each variable in two conditions: Vision and No-Vision. Each variable was computed for APA, flexion, switch, and extension phases based on the motion phase. The indicators derived from the analysis of this study include the maximum COP-AP displacement in the APA phase, the maximum COP-AP displacement during flexion, the duration of each motion phase, the maximum trunk flexion angle, and the angular velocity at each phase. In terms of muscle activity, the mean back muscle activity (ES), the mean abdominal muscle activity (RA), and the mean CCI were calculated in each motion phase. EEG calculated the α/β ratio in the Fp, Cz, and POz regions during each motion phase.

We used random forest analysis to classify the conditions with and without visual information for statistical analysis. The random forest can analyze accuracy by the area under the curve (AUC) and calculate variable importance. In the random forest analysis, we used the Gini impurity criterion and the AUC as the accuracy index. We listed the parameters used in the random forest analysis (Table 1). Next, we used random forest analysis to extract the top 10 variables for classifying the conditions with and without visual information. The selection of the top 10 variables was guided by previous research [21], as there is no established threshold for variable importance in random forest analysis. To compare the extracted variables between conditions, we used a Wilcoxon signed-rank test based on the study’s small sample size, and there was no visual confirmation of the normal distribution. The significance level was set at 5%. The R 4.2.3 software was used for statistical analysis.

## 3. Results

### 3.1. Variable Importance for Classifying Conditions with and without Visual Information Using Random Forest Analysis

**Figure 2:**
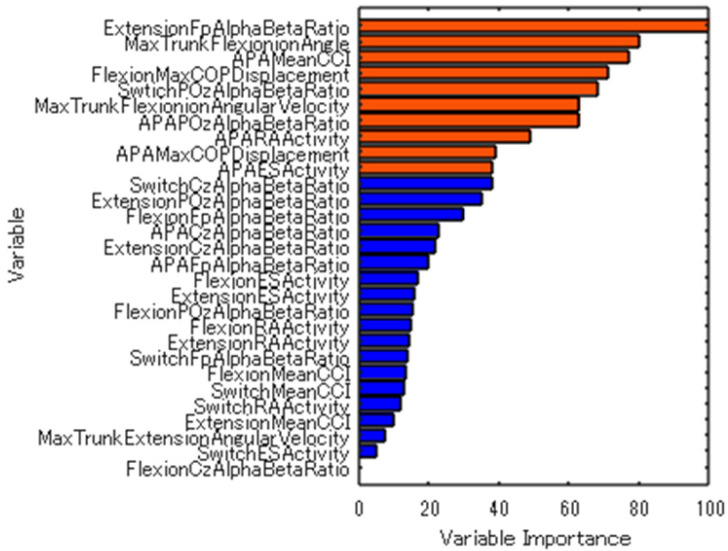
Variable importance for classifying conditions with and without visual information. Max: maximum; APA: anticipatory postural adjustments; COP: center of pressure; RA: rectus abdominis; ES: erector spinae; CCI: co-contraction index.

The variable importance for classifying the conditions with and without visual information was calculated using random forest analysis (Figure 2). The top 10 variables with highest importance were extracted, revealing that the α/β ratio in the Fp region during the extension phase, the maximum trunk flexion angle, the maximum CCI in the APA phase, the maximum COP-AP displacement during trunk flexion movement, the α/β ratio in the POz region during the switch phase, the maximum angular velocity of the trunk flexion, the α/β ratio in the POz region during the APA phase, the mean RA activity during the APA phase, the maximum COP-AP displacement in the APA phase, and the mean ES activity during the APA phase were extracted. The model’s accuracy index was area under the curve (AUC) = 0.84.

### 3.2. Comparison of Variables between Vision and No-Vision Conditions

For the top 10 variables extracted, two-group comparisons were conducted to compare the conditions (Table 2). For the kinematic and kinetic indices, the maximum flexion angle was lower in the No-Vision condition than in the Vision condition (95% CI: 2.5 to 3.6; *p* < 0.001, r = 0.87). Additionally, the maximum trunk flexion angular velocity was faster in the No-Vision condition (95% CI: −12.0 to −3.0; *p* < 0.001, r = −0.61). For the COP indices, the maximum forward displacement during flexion movement was smaller in the No-Vision condition (95% CI: 4.5 to 14.1; *p* < 0.001, r = 0.59), and although there was no significant difference in the maximum displacement during the APA phase, there was a tendency for it to be smaller (95% CI: −2.3 to 0.8; *p* = 0.35, r = −0.16).

For the muscle activity indices, the mean RA activity in the APA phase (95% CI: −96.0 to 37.9; *p* = 0.59, r = 0.06), the mean ES activity in the APA phase (95% CI: −12.8 to 11.3; *p* = 0.91, r = 0.01), and the mean CCI in the APA phase (95% CI: −0.03 to 0.07; *p* = 0.86, r = 0.02) showed no significant difference.

**Figure 3:**
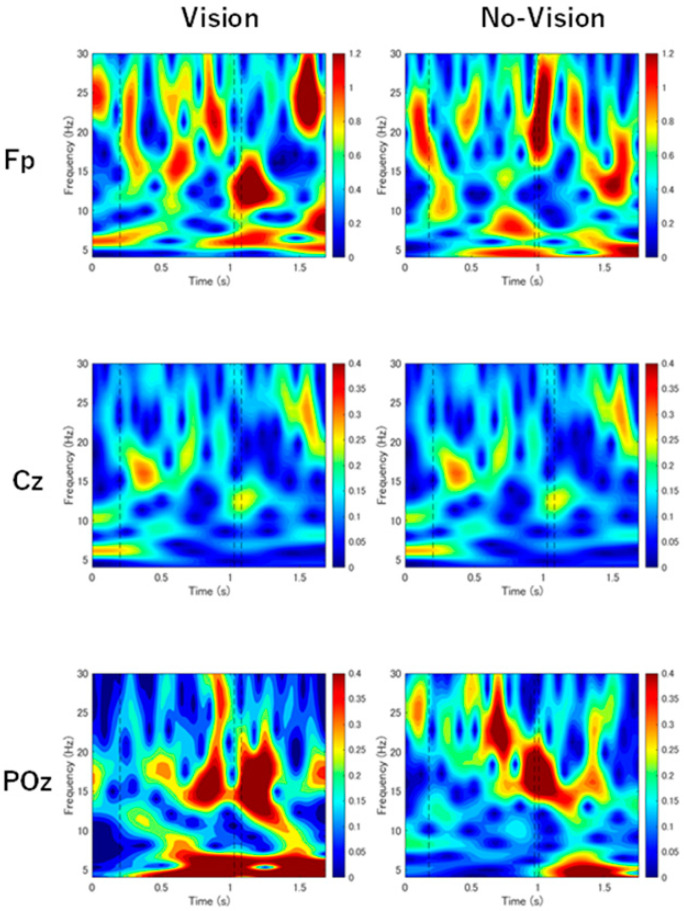
Scalograms of EEG for each channel in the Vision and No-vision conditions. The scalograms are from one repetition of one subject’s movement tasks. The Fp panels indicate the average of Fp1 and Fp2. The closer the color is to red, the higher the power value in the frequency band; the closer to blue, the lower the power value in the frequency band. Black lines in the figure indicate APA offset (flexion onset), flexion offset, and extension onset, from left to right.

For the EEG activity indices (Figure 3), the α/β ratio of the POz during the switch phase was higher in the No-Vision condition (95% CI: −0.4 to −0.002; *p* = 0.047, r = −0.34). The α/β ratio of the POz in the APA phase (95% CI: −0.1 to 0.07; *p* = 0.57, r = −0.10) and the α/β ratio of the Fp in the extension phase (95% CI: −0.7 to 0.03; *p* = 0.078, r = −0.30) showed no significant difference, but there was a tendency for the No-Vision condition to show higher values. 

## 4. Discussion

This study investigated the impact of sensory re-weighting due to visual deprivation on dynamic posture control indicators in trunk movements. It examined neurophysiological mechanisms by analyzing brain activity. Using a random forest analysis for variable importance, movement angle, movement angular velocity, COP-AP, muscle activity, and EEG activity indicators were extracted as variables influencing the classification of the Vision and No-Vision conditions. Comparing the extracted indicators between the conditions showed that the No-Vision condition resulted in lower maximum forward displacement than the Vision condition for COP-AP. Regarding the movement analysis, the maximum flexion angle was lower, and the maximum flexion angular velocity was higher in the No-Vision condition than in the Vision condition. For EEG activity, the α/β ratio of POz during the switch phase was higher in the No-Vision condition than in the Vision condition.

Previous research focusing on kinematic and kinetic indicators reports that posture perturbations that occur below the vestibular sensory detection threshold do not affect the upright posture of healthy young adults when vision is absent [22]. Additionally, when focusing on static posture control when visual information is reduced, it has been reported that the body sway in the anterior–posterior direction is more affected than that in the lateral direction [23]. However, the influence of closing the eyes during movement on COP-AP displacement has not been clarified. Previous research investigated the effect of position on the perception of trunk flexion angles when standing with the eyes closed, reporting that participants underestimated trunk flexion angles when the starting position was close to a quiet standing posture, overestimated them when it was close to maximum trunk flexion, and correctly recognized them at intermediate positions [24]. Additionally, when focusing on movement angle/angular velocity in spinal stability, it has been reported that spinal stability improves when the trunk flexion angle is large and movement is fast [25]. Based on prior findings, this may suggest that the participants overestimated the trunk flexion angle in the No-Vision condition, which may explain why the maximum flexion angle was lower. The maximum flexion angular velocity was higher in the No-Vision condition than in the Vision condition; this may have been due to the higher flexion angular velocity improving spinal stability. 

In muscle activity, there was no significant difference between the Vision and No-Vision conditions. In this study, the RA mean muscle activity, ES mean muscle activity, and mean CCI ratio in the APA phase influenced the classification of the Vision and No-Vision conditions. Prior research has reported that visual information affects the muscle activity of the abdominal and back muscles during anticipatory posture control [11]. Additionally, it is reported that vision not only provides information about the timing of perturbations, but also enables predictive reactions to perturbations through unconscious neuromuscular pre-activation and appropriate activation [11]. Thus, based on prior findings, the ability to anticipate reactions to trunk flexion movements due to visual information may be related to the extracted muscle activity indicators for APA phases as important variables for classifying conditions with and without visual information.

In terms of EEG, this study showed specific activity in the POz region in the APA and switch phases, and in the Fp region in the extension phase under visual deprivation, compared with the visual condition. The sensory organization test (SOT) is used to evaluate the ability to adjust sensory weighting, depending on the presence or absence of visual information [26]. SOT is an evaluation method that sets six conditions that systematically disrupt sensory integration processes in proprioceptive or visual inputs while measuring the subject’s ability to maintain balance. Using the six sensory conditions, evaluating the relative contributions of visual, vestibular, and somatosensory inputs in balance function is possible. In this study, as in the SOT, it was assumed that sensory weighting was adjusted to rely on proprioceptive and vestibular sensory information due to the deprivation of visual information. The POz region, extracted as a significant brain-wave region for classifying conditions with and without visual information, corresponds to the superior parietal lobule. In contrast, the Fp region corresponds to the prefrontal cortex. The superior parietal lobule is considered a region related to sensorimotor integration [26,27]. It is reported that the superior parietal lobule plays an essential role in realizing high-level cognitive functions, such as the perception of behavior, by integrating information from vision, movement, and somatosensory areas [27]. In this study, the α/β ratio in the POz region in the APA and switch phases was associated with classifying the Vision and No-Vision conditions. The APA phase is the preparatory phase for anticipatory postural control in the initiation of trunk flexion, and the switch phase is the period for switching the direction of movement between flexion and extension. These are essential phases for realizing cognitive functions related to behavior. Activation of the superior parietal lobule is associated with planning, executing, and sensing tasks that require anticipatory activation of postural muscles in the trunk- and hip-muscle tissue [28]. The superior parietal lobule also assists sensorimotor transformation as a sensorimotor hub for interacting with the environment [27]. It has a multifaceted role in spatial processing, encoding various types of information about body movements, such as functional body positions and vector information on movement [29]. Therefore, in the APA and switch phases, the α/β ratio in the POz region was extracted as an important variable for classifying the Vision and No-Vision conditions due to its role in sensorimotor transformation in anticipatory posture control and spatial processing.

Focusing on the α/β ratio in the Fp region during the extension phase, previous research reported increased prefrontal cortex activity during backward stepping compared to forward stepping [30]. Backward stepping requires enhanced cognitive control and is considered avoidance behavior. It is also reported that the prefrontal cortex is associated with exploratory behavior [31,32]. Typically, movement in the extension direction cannot receive input from the visual system, and compared to movement in the flexion direction, the extension direction is more dependent on proprioception. Thus, the need to exert exploratory cognitive control by increasing prefrontal cortex activity is suggested. In this study, although no significant difference was observed between the Vision and No-Vision conditions, the moderate effect-size difference suggests that the α/β ratio in the Fp region during the extension phase was extracted as an important variable for classification.

This study is the first to comprehensively investigate the impact of sensory re-weighting on trunk motor control under visual deprivation. Its unique point is that it incorporated machine learning methods and used kinematic and kinetic indicators, muscle activity, and brain waves. In human posture control, sensory cues are crucial, and when one intends to move, changes in perceptual information, such as vision, vestibular sensory information, and somatosensory perception, are expected. The comparison of predicted sensory information with actual feedback sensory information achieves posture control through sensory integration [33]. This study focused on re-weighting sensory information, including vision, vestibular sensory information, and somatosensory perception, based on changes in visual information. In posture control, sensory integration is also based on sensory re-weighting according to the sensory cues available [34]. The results of this study suggest that the sensory re-weighting due to reliance on proprioceptive and vestibular sensory information, induced by visual deprivation, influenced the motor control indicators in trunk flexion–extension movements through sensorimotor transformation in the superior parietal lobule and exploratory cognitive control in the prefrontal cortex. Considering that the contribution of proprioceptive sensory information is high in sensory information involved in postural control [35] and that vestibular sensory suppression occurs under head movement [36], such as in the present study, these results indicate that sensory re-weighting with a predominance of proprioceptive sensation was applied in the trunk movement task under visual deprivation. Our study suggests using visual deprivation to evaluate the sensory integration function focused on sensory re-weighting proprioception and vestibular sensory information in the trunk.

This study has some limitations. First, the participants were limited to healthy young adults. Age differences or the presence of pain could lead to differences in characteristics of brain-wave activity, muscle activity, and kinematic indicators, which limits generalizability. Second, the study focused on proprioception’s weighting in posture control through visual deprivation. However, vestibular sensory information weighting might have also occurred due to visual deprivation, which limits the investigation to proprioception alone. Third, the EEG measurement sites in this study were limited to Fp1, Fp2, Cz, and POz, which means that other sites were not tested. The premotor cortex (FCC3, FCC4) and supplementary motor area (FCz, Fz), which were not measured in this study, are involved in external motor programming [37], probably playing a role in motor program modification. Fourth, the functional connectivity between the EEG measurement sites was not evaluated, making the determination of relationships between brain function areas difficult. Fifth, the comprehensive relationship between brain waves, kinematic indicators, and muscle activity was not assessed. Future research needs to investigate different types of participants, different EEG activity measurement sites, and the relationship between measurement indicators to evaluate the impact of proprioceptive weighting on dynamic posture control in clinical practice.

## 5. Conclusions

This study suggests that the effect of sensory re-weighting on trunk motor control under visual deprivation influences kinematic/kinetic indicators, muscle activity, and brain-wave activity in trunk flexion–extension movements. The specific influence of cognitive- and sensory-integration-related brain regions on motion phases suggests that information processing involving sensory integration and cognition, including sensory re-weighting induced by visual deprivation, affects movement control.

## Figures and Tables

**Table 1:** The parameters used in the random forest analysis.

Kinematics/Kinetics index
Overall maximum trunk flexion angle
Overall maximum trunk flexion angular velocity
Overall maximum trunk extension angular velocity
Overall maximum COP-AP displacement during APA phase
Overall maximum COP-AP displacement during trunk flexion movement
EMG index
APA phase RA mean muscle activity
Flexion phase RA mean muscle activity
Switch phase RA mean muscle activity
Extension phase RA mean muscle activity
APA phase ES mean muscle activity
Flexion phase ES mean muscle activity
Switch phase ES mean muscle activity
Extension phase ES mean muscle activity
APA phase mean CCI
Flexion phase mean CCI
Switch phase mean CCI
Extension phase mean CCI
EEG index
APA phase Fp α/β ratio
Flexion phase Fp α/β ratio
Switch phase Fp α/β ratio
Extension phase Fp α/β ratio
APA phase Cz α/β ratio
Flexion phase Cz α/β ratio
Switch phase Cz α/β ratio
Extension phase Cz α/β ratio
APA phase POz α/β ratio
Flexion phase POz α/β ratio
Switch phase POz α/β ratio
Extension phase POz α/β ratio

COP: center of pressure, AP: anterior–posterior, APA: anticipatory postural adjustments, CCI: co-contraction index.

**Table 2:** Characteristics of the top 10 important variables in the Vision and No-Vision conditions.

	Vision	No-Vision	*p*-Value
**Kinematics/Kinetics index**			
Maximum trunk flexion angle (°)	48.8 (44.7–57.5)	46.6 (41.0–53.8)	<0.001
Maximum trunk flexion angular velocity (degree/s)	80.5 (66.6–96.1)	89.7 (69.6–113.6)	<0.001
Maximum COP-AP displacement during APA phase (mm)	7.0 (4.4–10.6)	6.6 (4.5–10.6)	0.35
Maximum COP-AP displacement during trunk flexion movement (mm)	47.4 (33.8–67.0)	35.0 (22.6–55.2)	<0.001
EMG index			
APA phase RA mean muscle activity (%)	152 (111–244)	142 (112–196)	0.59
APA phase ES mean muscle activity (%)	645 (518–785)	633 (507–797)	0.91
APA phase mean CCI ratio	0.5 (0.4–0.5)	0.5 (0.4–0.5)	0.86
EEG index			
APA phase POz α/β ratio	1.0 (0.9–1.2)	1.1 (0.9–1.3)	0.57
Switch phase POz α/β ratio	1.0 (0.8–1.1)	1.1 (0.9–1.4)	0.047
Extension phase Fp α/β ratio	1.0 (0.7–1.3)	1.1 (0.9–1.8)	0.078

Median (interquartile range). COP: center of pressure, AP: anterior–posterior, RA: rectus abdominis, ES: erector spinae, APA: anticipatory postural adjustments, CCI: co-contraction index.

## Data Availability

The data presented in this study are available on request from the corresponding author. The data are not publicly available due to privacy and ethical restrictions.
